# Emergency hybrid surgery for transection of pancreas at the head and neck after blunt abdominal trauma: A case report and review of the literature

**DOI:** 10.1097/MD.0000000000037144

**Published:** 2023-02-02

**Authors:** Yanan Xu, Tao Ai

**Affiliations:** a Department of Traumatology, Chongqing Emergency Medical Center/Chongqing University Central Hospital, Chongqing, China.

**Keywords:** blunt pancreatic trauma, case report, endoscope, main pancreatic duct, stent placement

## Abstract

**Introduction:**

A complete disruption of main pancreatic duct (MPD) presents a significant challenge to the surgeon. Historically, the standard surgical approach for addressing a complete disruption of the MPD involved distal pancreatic resection and pancreaticojejunostomy Roux-en-Y anastomosis. Nevertheless, there have been no reported cases of hybrid surgery being employed for the complete disruption of the MPD.

**Patient concerns:**

A 63-year-old male patient presented with blunt trauma in the upper abdomen and was transferred to our trauma center 10 hours after injury. Upon arrival at the emergency department, he was conscious, hemodynamically stable, and complained of upper abdominal pain and distention. Physical examination revealed right upper abdominal tenderness and slight abdominal tension. Abdominal contrast-enhanced CT scan revealed a complete transection of pancreatic parenchyma at the junction of the head and neck.

**Diagnoses:**

Complete transection of pancreatic parenchyma at the junction of the head and neck combined with complete disruption of the MPD, AIS grade IV.

**Interventions:**

The hybrid surgery was initially utilized for complete MPD disruption, incorporating endoscope-assisted stent placement in the MPD along with primary repair of the pancreatic parenchyma and duct.

**Outcomes:**

The postoperative period went smoothly, and the patient recovered and was discharged 4 weeks after operation. The MPD stent was removed under endoscope 4 months after operation, and Endoscopic Retrograde Pancreatography examination showed that the MPD was patency and slight MPD stenosis without pancreatic leakage. At the most recent follow-up, the patient had returned to normal life and work without any pancreatic endocrine or exocrine dysfunction.

**Lessons:**

The hybrid surgery, incorporating endoscope-assisted MPD stent placement and primary repair of the pancreatic parenchyma and duct, emerges as a promising alternative for complete MPD disruption in hemodynamically stable patients. The challenge in this hybrid surgery is the precise localization of the distal end of the MPD.

## 1. Introduction

Blunt pancreatic injuries, often resulting from acceleration-deceleration forces and direct compression in the upper abdomen, and are relatively rare, comprising only 0.25% of all trauma cases, 2.4% of abdominal traumas, and contributing to 4.3% of mortality rates.^[1]^ The main pancreatic duct (MPD) is particularly susceptible to injury in these cases, with an incidence as high as 62%, possibly due to its greater fragility compared to adjacent blood vessels and parenchyma.^[2]^ The MPD injury not only is the major determinant of pancreatic treatment modality and pancreas-related complications but also determines the severity of blunt pancreatic injury.^[3–5]^ While non-surgical approaches and pancreatic duct stenting can suffice for normal or partially injured MPDs, complete MPD disruptions warrant surgical intervention.^[6]^ The choice of pancreatic surgical procedure depends on the location of the transection of the pancreatic injury. In the past, the standard surgical procedure included distal pancreatic resection, proximal pancreatic closure, and distal pancreaticojejunostomy Roux-en-Y anastomosis.^[7]^ More recently, bridge stenting along the injured MPD has been employed with success.^[8]^ However, limited cases have been reported on hybrid surgery- endoscope-assisted MPD stent placement and primary repair of the pancreatic parenchyma and duct for pancreatic neck injuries. In this study, we present a case of a 63-year-old male patient who underwent hybrid surgery to address a complete transection of the pancreas at the head and neck. Ethical approval for this study was obtained from the ethics committee of the Chongqing Emergency Medical Center. The patient and his family were informed about abdominal emergency hybrid surgery procedure and relevant medical documents were signed after obtaining their consent.

## 2. Methods and results

**Figure 1.**
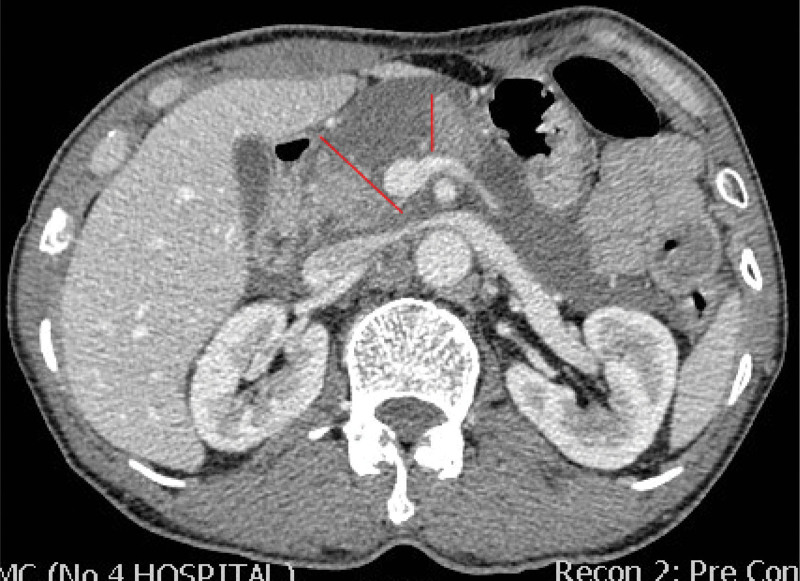
Emergency abdominal contrast-enhanced computed tomography showing transection (between red line) of pancreatic head and neck at the right of the superior mesenteric vein, suggestive of complete disruption of the MPD. MPD = main pancreatic duct.

A 63-year-old male patient presented with blunt trauma resulting from hitting in the upper abdomen by a heavy object and was transferred to our trauma center 10 hours after injury. Upon arrival, he remained conscious and hemodynamically stable, but complained of upper abdominal pain and distention. Physical examination revealed right upper abdominal tenderness, slight abdominal tension, and no rebound pain. Blood tests indicated serum amylase and lipase levels were 574 and 438.3 unit per liter (U/L), respectively. Contrast-enhanced computed tomography scan of the abdomen revealed hematoma around the pancreatic neck and the intestinal space in the right upper abdomen, complete transection of pancreatic parenchyma at the junction of the head and neck, suggestive of MPD disruption (Fig. 1). The disruption was located on the right of the superior mesenteric vein.

**Figure 2.**
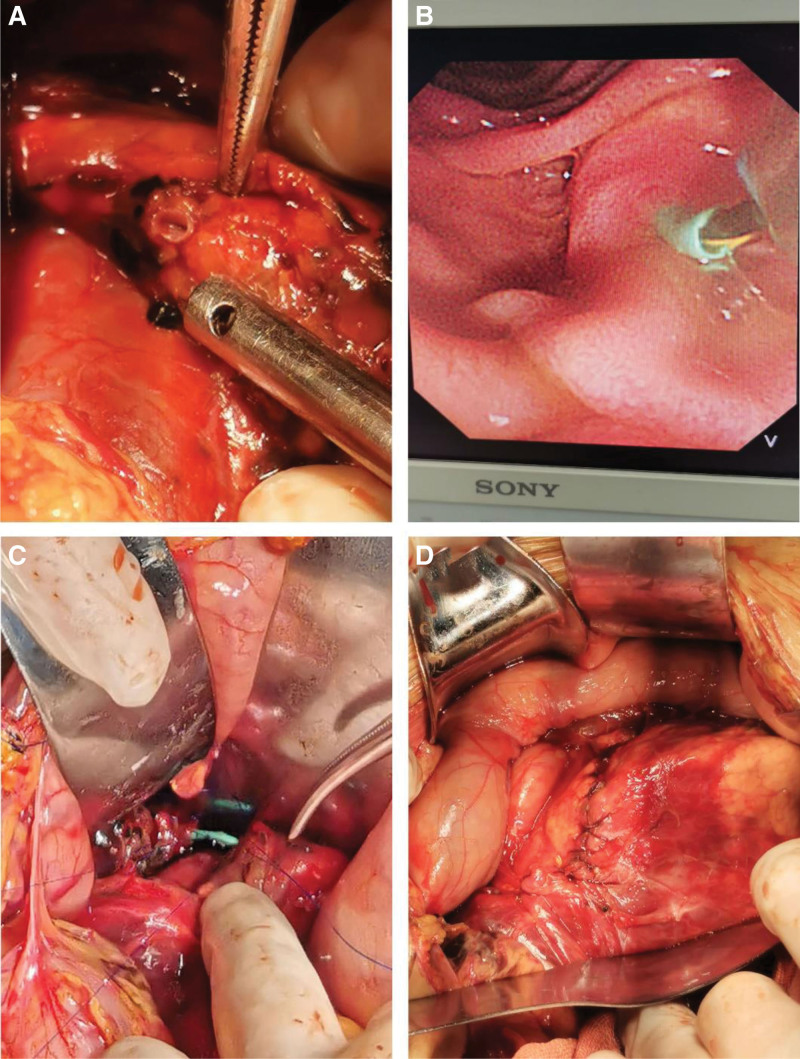
Emergency surgical procedure. Note: (A) Severed end of MPD. (B) Implantation stent under endoscopic guidance. (C) Duct-to-duct anastomosis of the MPD with the stent as structural support. (D) End-to-end anastomosis of the pancreatic parenchyma. MPD = main pancreatic duct.

We performed endoscope-assisted stent placement of the MPD and primary repair of the pancreatic parenchyma and duct due to the patient hemodynamic stability, the absence of any concomitant injuries, and recognization of the proximal and distal ends of MPD easily. During laparotomy, we observed complete transection of the pancreatic head and neck at the right of the superior mesenteric vein, with no signs of local inflammation or substantial necrosis. Both proximal and distal ends of the MPD were easily identified, displaying normal size with a diameter exceeding 2 mm. After the proximal and distal ends of the MPD were found, the duodenoscope was inserted by the gastroenterologists and the 0.035-inch guidewire was passed through the duodenal papilla. When the guidewire was passed through the proximal end of the MPD, the surgeon manipulated the guidewire tip to place it into the distal end of the MPD. A plastic stent (7F and 5 cm) was deployed through the guidewire by the gastroenterologists under endoscopic guidance. With the stent as structural support, a duct-to-duct anastomosis was performed for MPD with Prolene 4-0, and the pancreatic parenchyma was end-to-end anastomosed intermittently with 4-0 polydioxanone suture. Next, we placed 8 tubes in the abdominal cavity, including 2 pancreatic bed drainage tubes, 1 pancreatic bed irrigation tube, 1 jejunal nutrition tube and 1 gastrostomy tube, perihepatic, splenic fossa, and pelvic drainage tubes, in which the pancreatic bed drainage tubes were placed above and below the pancreas, respectively (Fig. 2).

The amylase content in pancreatic bed drainage fluid was 32,110 U/L after the operation indicating that end-to-end anastomosis of the MPD and parenchyma could not prevent pancreatic leakage. On postoperative day 1, the pancreatic bed was continuously irrigated with 3000 mL of normal saline for 24 hours daily. After rinsing, a white floccule was discovered in the pancreatic bed drainage tube, and the rinsing fluid was a little cloudy, declaring that rinsing could flush out the necrotic tissue and pancreatic fluid. The amylase content of peritoneal drainage decreased to 220 U/L after irrigation, indicating that irrigation could significantly reduce the amylase content around the pancreas and significantly decreased complications such as peripancreatic abscesses. No fluid was extracted from the perihepatic, splenic, and pelvic drainage tubes after operation, indicating that irrigation would not cause the diffusion of pancreatic fluid around the abdominal cavity. Serum amylase and lipase decreased to 168 and 203.1 U/L respectively on postoperative day 2, indicating a good operation effect. On the 3rd postoperative day, enteral nutrition support therapy was started with a jejunal nutrition tube. The postoperative period went smoothly, and the patient recovered well and was discharged 4 weeks after operation. The MPD stent was removed under endoscope 4 months later, and Endoscopic Retrograde Pancreatography examination showed that the MPD was patency and slight MPD stenosis without pancreatic leakage. At the most recent follow-up, the patient had returned to normal life and work without any pancreatic endocrine or exocrine dysfunction and was very satisfied with the overall treatment result.

## 3. Discussion

According to current researches, the choice of treatment for pancreatic injury depends mostly on hemodynamic stability, MPD integrity and the injury location of MPD.^[7,9]^ An unstable patient is more likely to undergo early laparotomy for damage control during which the pancreas can be evaluated. On the other hand, a stable patient can be initially managed conservatively and definite treatment decisions can be made based on further diagnostic assessments.^[9,10]^ Non-surgical treatment is often recommended for grade 1 to 2 pancreatic injuries, while complete MPD ruptures typically necessitate surgical intervention.^[11,12]^ Although many authors attempted endoscopic stenting to treat pancreatic injury in recent years, it was only for patients with normal or partial damage of the MPD.^[13]^ For patients with complete injuries of the MPD, surgical options mainly include distal pancreatic resection, proximal pancreatic closure and distal pancreaticojejunostomy Roux-en-Y anastomosis, and Whipple surgery.^[11]^ However, these operations not only require longer operation time and more intraoperative blood loss but also disrupt normal bowel.

In recent years, some authors have attempted to directly support the MPD with stents for pancreatic duct anastomosis and achieved good results.^[8,14,15]^ Since stents were placed under direct vision during surgery as reported in the literature,^[8,10,14]^ it was not easy to place stents with a small diameter of MPD. Even if the diameter of MPD is normal, small-diameter stents should be selected for convenient placement. However, such selection may lead to the risk of stent obstruction or migration, which may lead to treatment failure and the possibility of late complications such as ductal stricture.^[2,6]^ Currently, endoscopic treatment of pancreatic injury is primarily employed for stenting in patients with normal or partial rupture of the MPD,^[2]^ or treatment of late complications such as pancreatic leakage and pseudocyst of the pancreas. It has been reported in the literature that endoscopic treatment for patients with complete rupture of the MPD carries a high risk of complications and mortality.^[13]^ We attempted for the first time to perform hybrid surgery for grade IV blunt pancreatic trauma, which installed the MPD stent with the assistance of endoscope during operation, and then performed end-to-end anastomosis of the MPD and pancreatic parenchyma. Compared with endoscopic stent therapy,^[13]^ the surgeon manipulated the guidewire to pass through the distal end of MPD under direct vision in hybrid surgery which can not only reduce the difficulty of catheterization and improve the success rate of catheterization, but also reduce various complications caused by repeated catheterization. Compared with traditional pancreatectomy,^[12]^ this method can effectively avoid excision of excessive pancreatic tissue, which may lead to internal and external secretion dysfunction. Moreover, the operation time of hybrid surgery is shorter and the intraoperative bleeding is less. Compared with intraoperative stent placement by the surgeon,^[8,10,14]^ the hybrid surgery is easier to place stents to the proximal and distal ends of the MPD by a guidewire with the assistance of endoscope, and a relatively bigger stent can be selected which can effectively avoid MPD stenosis after anastomosis. The challenge in this hybrid operation lies in locating the distal end of the MPD. In another case of pancreatic injury, we planned to reattempt this method; however, due to the inability to locate the distal end of the MPD, we had to abandon stent implantation and choose distal pancreatic resection instead. Current reports indicated the use of intraoperative ultrasound to successfully locate the MPD.^[16,17]^ If intraoperative ultrasound can consistently assist in locating the MPD, it may become a standard procedure for treating pancreatic injuries combined with complete disruption of the MPD using this hybrid surgery.

In this case, the MPD was successfully reconstructed by hybrid surgery, and there were no postoperative complications such as post-traumatic pancreatitis caused by stenosis of the MPD, and the long-term follow-up proved that the operation achieved good results. Pancreatic leakage is inevitable after pancreatic operation. While continuous lavage and drainage of the pancreatic bed with normal saline through the intraoperative preset irrigation tube in the early postoperative period can avoid pancreas-related complications, such as corrosive bleeding, pancreatic abscess, pancreatic intestinal fistula, etc. The main purpose of pancreatic bed lavage at the early postoperative stage was to dilute pancreatic fluid so that pancreatic enzymes in the leakage could be reduced to a harmless concentration without causing any complications. Therefore, continuous uniform lavage rather than intermittent lavage was adopted for 24 hours. The concentration of pancreatic enzyme in the peritoneal lavage was monitored daily. Postoperative outcomes also confirmed that irrigation would not cause pancreatic fluid to spread around the abdominal cavity, leading to other infections and intestinal fistula, because even if the rinse fluid was diffused, it would not cause related secondary complications because of the low content of pancreatic amylase. In addition, drainage tubes were placed at each low point of the abdominal cavity during the operation to ensure timely drainage of the effusion. The selection of pancreatic surgical methods determines the occurrence of postoperative complications, while careful intraoperative management can significantly reduce or alleviate postoperative complications.

## 4. Conclusions

The hybrid surgery, incorporating endoscope-assisted MPD stent placement and primary repair of the pancreatic parenchyma and duct, emerges as a promising alternative for complete MPD disruption in hemodynamically stable patients. The challenge in this hybrid surgery is the precise localization of the distal end of the MPD. Continuous lavage of the pancreatic bed in the early postoperative period plays a vital role in reducing pancreas-related complications.

## Acknowledgments

We are very grateful to Dr Peng Peng, deputy chief physician of the Department of Gastroenterology, for providing endoscopic technical support during the operation. This report is published with the knowledge and consent of the patient. We are very grateful to the patient Wu for his support and trust in our hospital.

## Author contribution

**Project administration:** Yanan xu, Tao Ai.

**Writing – original draft:** Yanan Xu.

**Writing – review & editing:** Yanan Xu.
